# Proteins derived from neutrophil extracellular traps may serve as self-antigens and mediate organ damage in autoimmune diseases

**DOI:** 10.3389/fimmu.2012.00380

**Published:** 2012-12-14

**Authors:** Jason S. Knight, Carmelo Carmona-Rivera, Mariana J. Kaplan

**Affiliations:** Division of Rheumatology, Department of Internal Medicine, University of Michigan Medical SchoolAnn Arbor, MI, USA

**Keywords:** neutrophil, NETs, autoimmunity, posttranslational modifications, systemic lupus erythematosus (SLE), psoriasis, vasculitis, citrullination

## Abstract

Neutrophils are the most abundant leukocytes in circulation and represent one of the first lines of defense against invading pathogens. Neutrophils possess a vast arsenal of antimicrobial proteins, which can be released from the cell by a death program termed NETosis. Neutrophil extracellular traps (NETs) are web-like structures consisting of decondensed chromatin decorated with granular and cytosolic proteins. Both exuberant NETosis and impaired clearance of NETs have been implicated in the organ damage of autoimmune diseases, such as systemic lupus erythematosus (SLE), small vessel vasculitis (SVV), and psoriasis. NETs may also represent an important source of modified autoantigens in SLE and SVV. Here, we review the autoimmune diseases linked to NETosis, with a focus on how modified proteins externalized on NETs may trigger loss of immune tolerance and promote organ damage.

## Introduction

Neutrophils are the most abundant leukocyte population in peripheral blood and have a lifespan of as little as 4 h in the circulation; this short half-life is balanced by continuous and tightly regulated release from the bone marrow. Neutrophils are among the first line of defense against invading microbes (Kobayashi and Deleo, 2009), targeting pathogens through diverse mechanisms including phagocytosis, reactive oxygen species (ROS) generation, the release of microbicidal molecules from cytoplasmic granules, and the recently described extrusion of an extracellular chromatin meshwork—so-called NETosis (Brinkmann et al., 2004).

In 2004, Brinkmann et al. described a distinct mechanism of neutrophil cell death, resulting in the programmed externalization of a meshwork of chromatin fibers decorated with granule-derived antimicrobial proteins (neutrophil extracellular traps or NETs) (Brinkmann et al., 2004). NETosis has subsequently been shown to be an important strategy by which neutrophils trap and kill invading microorganisms (Brinkmann and Zychlinsky, 2012; Kaplan and Radic, 2012). NETs can also damage the vasculature and have the potential to trigger thrombosis (Fuchs et al., 2010; Gupta et al., 2010; Brill et al., 2012; Saffarzadeh et al., 2012).

Although there is still much to learn regarding the triggers and signaling pathways that facilitate NETosis, important roles have been suggested for the NADPH oxidase machinery (Fuchs et al., 2007; Ermert et al., 2009; Bianchi et al., 2011; Remijsen et al., 2011), ROS (Nishinaka et al., 2011; Palmer et al., 2012), the Raf/mitogen-activated protein kinase/extracellular signal-regulated kinase pathway (Hakkim et al., 2011), histone citrullination (Neeli et al., 2008; Wang et al., 2009; Li et al., 2010; Hemmers et al., 2011), MPO/neutrophil elastase (NE) (Papayannopoulos et al., 2010; Metzler et al., 2011), autophagy (Mitroulis et al., 2011; Remijsen et al., 2011), and microtubule polymerization (Neeli et al., 2009). The characterization of pathways implicated in the development of NETs has potential implications for pharmacologic strategies to block NETosis, which is particularly appealing in the context of the “sterile” NETosis that will be described below. The protein fraction of NETs classically contains histones, MPO, and various serine proteases, although the specific composition continues to be defined (Urban et al., 2009; Liu et al., 2012). Here, we will first review recent discoveries pertaining to how NETs may play a role in the pathogenesis of systemic autoimmune diseases, and will then consider the protein composition of NETs in more detail.

## Small vessel vaculitis

The first compelling link between NETs and autoimmunity was in 2009 with the characterization of NETosis in small vessel vasculitis (SVV) (Kessenbrock et al., 2009). SVV is a systemic autoimmune disease of unknown etiology, with disease flares that result in necrotizing inflammation of small blood vessels—especially targeting the kidneys, lungs, skin, and peripheral nerves. The majority of SVV patients have detectable anti-neutrophil cytoplasmic antibodies (ANCA) with specific reactivity against either proteinase 3 (PR3) or MPO. In addition to their important role in diagnosis, ANCA activate neutrophils *in vitro* (Chen and Kallenberg, 2009), and are sufficient to induce vasculitic disease in animal models (Xiao et al., 2002; Pfister et al., 2004). Kessenbrock and colleagues showed that NETs externalize PR3 and MPO, and, reciprocally, that ANCA (and specifically anti-PR3 antibodies) induce NETosis (Kessenbrock et al., 2009). Furthermore, MPO-DNA complexes, presumably derived from NETs, can be detected in the circulation, the levels of which track with SVV disease activity. In addition, extracellular DNA (co-localizing with histones, MPO, and PR3) was detected in kidney biopsies from the majority of SVV patients (Kessenbrock et al., 2009).

While the Kessenbock study, as well as one additional case report (Abreu-Velez et al., 2009), have hinted at an important role for NETs in the organ damage of SVV, more recent studies have begun to mechanistically explore the specialized role of NET proteins as autoantigens in SVV. To this end, Nakazawa and colleagues studied the drug propylthiouracil (PTU) which is a known inducer of anti-MPO autoantibodies and SVV in humans (Wada et al., 2002; Nakazawa et al., 2012). In the presence of PTU, phorbol 12-myristate 13-acetate (PMA)-induced NETs had an abnormal, globular conformation, which was relatively resistant to DNase I digestion (Nakazawa et al., 2012). When these PMA/PTU NETs were injected into rats, the animals not only developed anti-MPO autoantibodies, but also pulmonary capillaritis reminiscent of human vasculitic disease (Nakazawa et al., 2012). Whether the driving feature of autoimmunity was enhanced NET stability, differences in the tertiary structure of PTU NETs or modification of NET proteins such as MPO remains to be determined.

Another recent study provided a tantalizing link between NETs and adaptive immunity, demonstrating that NET proteins were preferentially uploaded into myeloid dendritic cells (mDCs) *in vitro*, an affect that was lost when the NET structure was dismantled with DNase (Sangaletti et al., 2012). Injection of the NET-loaded mDCs into mice resulted in anti-MPO autoantibodies and an autoimmune phenotype including glomerulitis, although the kidney histopathology was in some ways more reminiscent of lupus lesions than the typical pauci-immune disease of SVV. This study hints that NETs provide a unique, stimulatory microenvironment that can break normal immune tolerance, and thereby predispose to autoimmunity.

## Psoriasis

Psoriasis is a common inflammatory disease of the skin and is typically thought of as an autoimmune disease given the recognized importance of autoreactive T-cells. In psoriasis, local production of type I IFNs, such as IFNα, by plasmacytoid dendritic cells (pDCs) is an important upstream event in the activation of autoimmune T-cells (Nestle et al., 2005). pDCs are a specialized type of dendritic cell with unique, high-level expression of toll-like receptors (TLRs) 7 and 9, which recognize nucleic acids from viruses and other microbes—the result being robust expression of type I IFNs (Kadowaki et al., 2001). In 2007, Lande et al. identified the cationic NET protein cathelicidin/LL37 as a factor that binds and converts inert self DNA into a complex capable of activating pDCs (Lande et al., 2007); this leads to robust production of IFNα in psoriasis skin, with implications for driving autoimmunity. More recently, it has also been suggested that the combination of secretory leukocyte proteinase inhibitor (SLPI) and NE, both derived from NETs, can bind DNA and serve a similar role in converting self DNA into an activator of pDCs in psoriasis lesions (Skrzeczynska-Moncznik et al., 2012).

Interleukin-17 (IL-17) is a proinflammatory cytokine, linked to autoimmune diseases such as rheumatoid arthritis (RA), inflammatory bowel disease, and psoriasis (Wilson et al., 2007). Historically, production of IL-17 has been attributed to Th17-cells, and, indeed, both IL-17 mRNA and increased numbers of Th17-cells have been identified in psoriasis lesions (Kryczek et al., 2008; Lowes et al., 2008). A novel take on this story was the recent identification of extracellular traps from both mast cells and neutrophils as an important, and perhaps predominant, source of IL-17 in psoriasis lesions (Lin et al., 2011). Further, IL-23, a known activator of Th17 differentiation, can also stimulate mast cells to release extracellular traps decorated with IL-17 (Lin et al., 2011).

## Gout

Acute gout is a common, inflammatory arthritis driven by the deposition of monosodium urate (MSU) crystals in appendicular joints; a critical impetus for MSU deposition is elevated serum uric acid, which correlates with obesity, hypertension, diabetes, and other metabolic risk factors. Although gout is not a typical autoimmune disease, it shares the characteristic of acute, sterile inflammation; and, in recent years, the recognition of the potency by which anti-IL-1 agents can ameliorate gout flares has opened the door to what will surely be additional cytokine manipulation in the future.

Given the now well-recognized role of MSU crystals as activators of the NLRP3 inflammasome with resultant production of the pro-inflammatory cytokine IL-1β (Martinon et al., 2006), as well as the consistent documentation of neutrophilia in acute gout synovial fluid (Popa-Nita and Naccache, 2010), investigators have begun to address the extent to which NETs factor into gout pathogenesis. Indeed, MSU crystals, IL-1β, and both synovial fluid and serum from patients with acute gout, all stimulate neutrophils to release NETs (Mitroulis et al., 2011). These “gout NETs” contain DNA, MPO, and the alarmin, high mobility group box chromosomal protein 1 (HMGB1), and may propagate the inflammatory response. Furthermore, the IL-1 inhibitor anakinra blocks NET release when control neutrophils are exposed to gout serum or synovial fluid (Mitroulis et al., 2011).

More recently, basophils and eosinophils (along with neutrophils) were been shown to release extracellular traps in response to MSU crystals. In contrast, monocyte-lineage cells, despite phagocytizing the crystals, did not release extracellular DNA (Schorn et al., 2012). The authors argued that MSU-induced NETs were qualitatively different from those induced by bacteria or PMA in that MSU NETs extended more “widely” in the culture plate, and were relatively resistant to inhibition (and perhaps degradation) by high concentrations of plasma in the culture medium (Schorn et al., 2012). The protein content of MSU-induced NETs was not, however, further explored.

## Felty's syndrome

Patients with RA—the prototypical chronic, inflammatory polyarthritis—form autoantibodies to citrullinated (deiminated) proteins, the detection of which has emerged as the most compelling serologic test for RA (Wegner et al., 2010). A small subset of patients with RA develop so-called Felty's syndrome, which manifests clinically as marked neutropenia and splenomegaly; and, which appears to be closely related to a syndrome of oligoclonal T-cell expansion, large granular lymphocyte leukemia (Balint and Balint, 2004; Liu and Loughran, 2011). Given the classical recognition of anti-histone antibodies in patients with systemic lupus erythematosus (SLE) (Suzuki et al., 1994), and to a lesser extent in RA and Felty's syndrome (Cohen and Webb, 1989; Tuaillon et al., 1990)—as well as the well-recognized deimination of histones in NETs (Neeli et al., 2008; Wang et al., 2009; Li et al., 2010; Hemmers et al., 2011)—a logical question is whether autoantibodies from SLE, RA, and Felty's syndrome patients specifically target deiminated histones.

In a 2012 study, autoantibodies from all three diseases showed reactivity with NETs, with a preference for deiminated histones in Felty's syndrome that was not readily apparent in either SLE or RA serum (Dwivedi et al., 2012). Further linking deimination to autoimmunity, deiminated histones were detected in circulating neutrophils of patients with RA, while serum from patients with SLE and Felty's syndrome stimulated the *ex vivo* deimination of neutrophil histones (Dwivedi et al., 2012).

## Systemic lupus erythematosus

SLE is an autoimmune syndrome characterized by autoantibody formation against nuclear antigens, with resultant immune complex deposition, inflammation, and organ damage (Tsokos, 2011). While intensive study has shown that both T- and B-cells are required for the lupus phenotype (Crispin et al., 2010; Dorner et al., 2011), neutrophils and other mediators of the innate immune response have, by comparison, received considerably less attention (Knight and Kaplan, 2012).

Various abnormalities in neutrophil phenotype and function have been described over the years, including abnormalities in phagocytic activity, aggregation, and intravascular activation (Brandt and Hedberg, 1969; Hashimoto et al., 1982; Abramson et al., 1983; Jonsson and Sturfelt, 1990; Molad et al., 1994; Courtney et al., 1999). Further, a subset of neutrophils in the peripheral blood of lupus patients have lower density and consequently co-purify with peripheral blood mononuclear cells (PBMCs) during sedimentation of whole blood (Hacbarth and Kajdacsy-Balla, 1986; Bennett et al., 2003; Denny et al., 2010). This population may represent the accelerated release of immature granulocytes from the bone marrow, although the origin, function, and pathogenic significance of these cells remain to be fully determined (Denny et al., 2010; Villanueva et al., 2011).

Evidence of a role for neutrophils in SLE pathogenesis is emphasized by the observation that various bactericidal proteins released by activated neutrophils are present at higher-than-expected levels in lupus blood (Sthoeger et al., 2009; Vordenbaumen et al., 2010; Ma et al., 2012). Neutrophils, and in particular low-density granulocytes (LDGs), have been associated with endothelial damage as well as promotion of abnormal endothelial differentiation, and have been posited to play a critical role in the well-recognized accelerated atherosclerosis of SLE (Denny et al., 2010; Kaplan, 2011). Neutrophilic infiltrates are a recognized feature of diffuse proliferative lupus nephritis (Austin et al., 1984), while proteins released from neutrophilic granules are toxic to glomerular structures (Henson, 1972; Johnson et al., 1988; Hotta et al., 1996).

A particularly exciting development of the past 2–3 years has been the description of aberrant NETosis in SLE, which might explain, at least in part, the longstanding recognition of increased circulating DNA in lupus patients (Tan et al., 1966). Indeed, mutations in DNase I have been reported among SLE patients, and seem to promote autoantibody formation (Yasutomo et al., 2001; Shin et al., 2004). In addition, two groups have recently described a DNase I-inhibititory activity in SLE serum that prevents degradation of NETs, and is associated with more active disease (Hakkim et al., 2010; Leffler et al., 2012). Specifically, experiments by Hakkim and colleagues demonstrate that 36.1% of SLE sera degrade NETs poorly, with inhibitors of DNase I detectable in some patients, while others coat the NETs with autoantibodies to mechanically protect against degradation (Hakkim et al., 2010). SLE patients with poor NET degradation have higher anti-double-stranded DNA antibody titers, display more complement activation, and are more likely to carry a diagnosis of lupus nephritis (Hakkim et al., 2010; Leffler et al., 2012).

**Figure 1:**
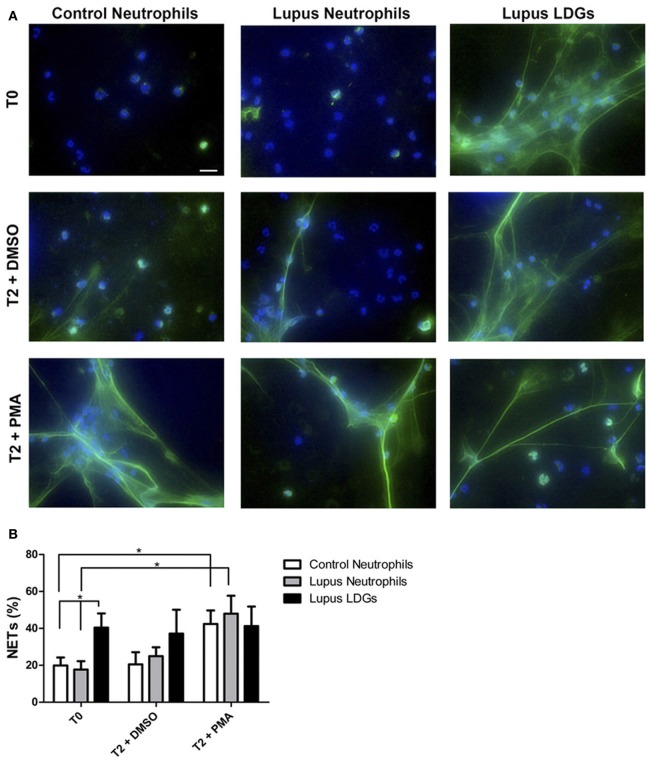
**Circulating lupus LDGs undergo increased NETosis. (A)** Representative images of control neutrophils, lupus neutrophils, and lupus LDGs isolated from peripheral blood and analyzed at baseline (T0) or after stimulation for 2 h with DMSO or PMA. Panels show merged images of neutrophil extracellular traps (NETs) in which neutrophil elastase is stained green by immunofluorescence and DNA is stained blue by Hoechst 33342; 40× images, scale bar: 20 μm. **(B)** Quantification of the percentage of NETs (elastase-labeled cells over total number of cells) are plotted as mean ± SEM (*n* = 6 patients/group; ^*^p = 0.05). [Obtained with permission from Villanueva et al. (2011) and The Journal of Immunology. Copyright 2011. The American Association of Immunologists, Inc.].

Further, in 2011, three papers described *ex vivo* models of enhanced NETosis in SLE patients (Garcia-Romo et al., 2011; Lande et al., 2011; Villanueva et al., 2011), with the aforementioned lupus LDGs particularly capable of releasing spontaneous NETs (Figure 1). All three papers also demonstrated that NETs stimulate pDCs to release type I IFNs (Garcia-Romo et al., 2011; Lande et al., 2011; Villanueva et al., 2011), and, indeed, most current models of lupus pathogenesis include a role for activation of the type I IFN pathway, which lowers the threshold for autoreactivity of both antigen-presenting and antibody-producing cells (Banchereau and Pascual, 2006; Elkon and Stone, 2011).

Continuing the theme discussed above for SVV and Felty's syndrome, NETs may provide novel antigens for autoantibody formation in SLE (Liu et al., 2012). There are also hints that NETs may be a source of vascular and organ damage in SLE (Villanueva et al., 2011), which would not be surprising if parallels were drawn to other inflammatory diseases where NET toxicity has been documented such as SVV, cystic fibrosis, transfusion-related acute lung injury (TRALI), and sepsis (Clark et al., 2007; Kessenbrock et al., 2009; Caudrillier et al., 2012; Dubois et al., 2012; Thomas et al., 2012).

## Net proteins and SLE

There are at least two general frameworks by which NET proteins might impact on SLE pathogenesis, both of which have already been touched upon in this review. The first posits a role for NETs in organ damage, which is supported by both the potential toxicity of NET proteins and the recognition that many of these proteins have been detected at increased levels in SLE patients. Proof of this principle will depend on animal models where specific proteins can be targeted by genetic or pharmacologic approaches.

The second concept is that NET proteins may be uniquely modified and positioned to break tolerance and thereby trigger or exacerbate autoimmunity. Certainly, the idea of modified proteins serving as autoantigens in SLE is not new (Casciola-Rosen et al., 1999; Utz et al., 2000; Graham and Utz, 2005; Dieker and Muller, 2010), and the milieu of NETs may represent a novel environment—replete with pathogens and immunostimulatory host molecules—where this can take place.

At this point, relatively few proteins have been definitively detected in lupus NETs, with the definition of “lupus NETs” being somewhat arbitrary and based on the *ex vivo* study of lupus neutrophils. Of the lupus-associated NET proteins, the most in-depth work has involved LL37/cathelicidin, with the demonstration that this small cationic protein circulates in complex with chromatin fragments and anti-DNA autoantibodies in lupus serum, thereby enhancing stimulation of pDCs and protecting against DNase-mediated destruction (Lande et al., 2011). Although not directly linked to SLE, LL37/cathelicidin can also complex with RNA to activate dendritic cells through TLR7 and TLR8 (Ganguly et al., 2009); this work is particularly interesting given the suggestion by Garcia-Romo and colleagues that anti-RNP antibodies and TLR7 may play a role not only in dendritic cell activation, but also in the activation of lupus neutrophils to release NETs (Garcia-Romo et al., 2011).

Other proteins identified by immunofluorescence (no lupus-NET proteomics have been undertaken outside of the histone studies described below) include C1q (Leffler et al., 2012), NE (Garcia-Romo et al., 2011; Villanueva et al., 2011), histones (Villanueva et al., 2011; Liu et al., 2012), HMGB1 (Garcia-Romo et al., 2011), HNP (Lande et al., 2011), IL-17 (Villanueva et al., 2011), LL37/cathelicidin (Lande et al., 2011; Villanueva et al., 2011), and MPO (Garcia-Romo et al., 2011; Lande et al., 2011; Villanueva et al., 2011). These proteins, along with those identified in the aforementioned autoimmune/inflammatory diseases, are summarized in Table 1. In the absence of a definitive proteomics approach, this list is certainly not exhaustive, and one might therefore extrapolate from other studies (Urban et al., 2009), with the caveat that the NETs heretofore characterized by proteomics were isolated from neutrophils treated with PMA, and therefore have unknown *in vivo* relevance from the perspective of SLE. We will now consider some of these individual lupus-NET proteins.

**Table 1:** **NET proteins with potential roles in autoimmunity**.

**Protein**	**Present in disease-specific NETs (by IF)**	Present in PMA-induced NETs (by proteomics^*^)	**AutoAbs**	**Role(s) in auto-immunity**
Histones	All (by definition)	Yes	SLE, Felty's	AutoAg in SLE and Felty's; pro-thrombotic
MPO	SLE, psoriasis, SVV, gout	Yes	SVV, SLE	AutoAg in SVV; oxidative stress?
Proteinase 3	SVV	Yes	SVV, SLE	AutoAg in SVV
LL37	SLE	No	SLE	Binds ICs to activate pDCs
HNP/α-defensins	SLE	Yes	SLE	Binds ICs; predisposes to CVD?
HMGB1	SLE, gout	No	Unknown	Binds ICs; pro-inflammatory
IL-17	SLE, psoriasis	No	SLE, psoriasis	Pro-inflammatory
C1q	SLE	No	SLE	Activates complement; protects from degradation?
Elastase	SLE, psoriasis	Yes	SLE	Unknown
Lactoferrin	Unknown	Yes	SLE	Unknown
Cathespin G	Unknown	Yes	SLE	Unknown
Calprotectin	Unknown	Yes	Unknown	Unknown
α-enolase	Unknown	Yes	SLE	Unknown
Catalase	Unknown	Yes	SLE	Oxidative stress?

* *(Urban et al., 2009); autoAb, autoantibody; autoAg, autoantigen; IC, immune complex*.

*NET, neutrophil extracellular trap; IF, immunofluorescence; SLE, systemic lupus erythematosus; SVV, small vessel vasculitis; CVD, cardiovascular disease*.

Both the peroxidase MPO and serine protease PR3 have compelling roles as autoantigens in SVV, as discussed above (Kessenbrock et al., 2009). And, given the common availability of commercial assays, anti-MPO and anti-PR3 titers have been frequently assessed in SLE patients (Nassberger et al., 1990; Cambridge et al., 1994; Manolova et al., 2001; Pan et al., 2008). The available data is heterogeneous and no clear trend has emerged, although one can be relatively confident in saying that—at least for the assays that are commercially available—anti-MPO/PR3 autoantibodies do not specifically identify SLE patients, nor do they track with specific disease manifestations. In contrast to the assessment of autoantibodies, studies that examine the role of the MPO protein in SLE are relatively limited, although at least one study has shown increased MPO plasma levels in lupus patients as compared to healthy controls (Telles et al., 2010), albeit without a clear correlation to disease activity. NE is also a recognized trigger of autoantibodies in SLE (Nassberger et al., 1989, 1990), but the clinical significance remains to be determined. In terms of the NE protein, one study has suggested higher plasma levels in SLE patients (Zhang et al., 1989).

Both the iron-chelator lactoferrin and the serine protease cathepsin G have been objectively identified in PMA-induced NETs (Urban et al., 2009), and both appear to function as autoantigens in SLE (Lee et al., 1992; Galeazzi et al., 1998; Zhao et al., 1998; Manolova et al., 2001; Caccavo et al., 2005); although, again, no clear clinical correlation has emerged. In terms of circulating protein, there is no correlation between plasma lactoferrin and either active or inactive SLE (Adeyemi et al., 1990; Tsai et al., 1991), while cathepsin G protein levels have not been considered.

Alarmins are endogenous mediators capable of enhancing innate and adaptive immune response through recruitment and activation of antigen-presenting cells. From the perspective of NET proteins, both the α-defensins (sometimes called neutrophil defensins or human neutrophil peptides/HNPs) and HMGB1 would be classified as alarmins. α-defensins 1 and 3 were identified in the proteomic analysis of PMA-induced NETs (Urban et al., 2009), while both HMGB1 (Garcia-Romo et al., 2011) and α-defensins/HNP (Lande et al., 2011) have been described in the context of lupus NETs.

α-defensins activate monocyte-lineage cells to release pro-inflammatory cytokines such as TNF-α and IL-1β; they also serve as chemokines for recruitment of diverse cell types including T-lymphocytes and dendritic cells; and can regulate activation of the complement cascade (Lehrer et al., 1993; Lehrer and Ganz, 2002). α-defensins have been linked to SLE both from the perspective of defensin-specific autoantibodies that correlate with disease activity (Tamiya et al., 2006), as well as circulating protein levels that seem to be higher in lupus patients (Sthoeger et al., 2009; Vordenbaumen et al., 2010); in fact, high α- and β-defensin levels were recently shown to correlate with cardiovascular disease in lupus patients (Vordenbaumen et al., 2012). One can certainly imagine a role for defensins in the induction of lupus inflammation and autoimmunity, and indeed this concept has been reviewed elsewhere (Froy and Sthoeger, 2009).

From a lupus perspective, HMGB1, a DNA-binding protein with alarmin potential when released into the extracellular space, has received considerable interest in recent years, as evidenced by the number of review articles written on this topic (Abdulahad et al., 2010; Pan et al., 2010; Urbonaviciute and Voll, 2011; Pisetsky, 2012). Initial reports described extracellular HMGB1 in cutaneous lesions (Popovic et al., 2005; Barkauskaite et al., 2007), but, more recently, this DNA-binding protein has been linked to lupus nephritis (Zickert et al., 2012)—where HMGB1 has been suggested as a novel urine biomarker for nephritis activity (Abdulahad et al., 2012). Similar to cathelicidin/LL37, HMGB1 associates with extracellular nucleosomes and potentiates their inflammatory potential through receptors such as TLR9 (Tian et al., 2007; Urbonaviciute et al., 2008). A recent review, however, points out that caution is necessary as HMGB1 function is critically-dependent on its redox state, and therefore detection may not always equate with pathologic potential (Pisetsky, 2012).

With the exception of the nuclear protein HMGB1, all of the aforementioned proteins are primarily derived from neutrophil granules. Cytoplasmic proteins such as the antimicrobial heterodimer calprotectin have been identified in NETs (Urban et al., 2009); the links between calprotectin and lupus are tenuous, although one study reported elevated circulating levels which, in a population of 100 patients, correlated with disease activity and anti-DNA autoantibodies (Haga et al., 1993). These findings have not been replicated in another study (Wahren et al., 1995). Autoantibodies to another NET protein, α-enolase (Urban et al., 2009)—which have gained notoriety for their possible association with Hashimoto's encephalopathy (Yoneda et al., 2007)—can also be detected in patients with SLE (Mosca et al., 2006). Similarly, autoantibodies to the NET protein catalase have been described in lupus (Mansour et al., 2008), with suggestion that these antibodies may be linked to oxidative stress.

To summarize, studies reporting autoantibodies to NET proteins are common in SLE, although with tenuous clinical correlations that have yet to be reproduced across studies; certainly, none of these autoantibodies are presently useful to the rheumatologist in clinic (with the possible exception of anti-histone antibodies). In contrast, some of the most clinically relevant autoantigens in SLE such as Ro, La, Smith, and RNP have yet to be identified in NETs (Villanueva et al., 2011). When circulating protein levels are considered, there is a trend toward NET proteins being increased in lupus plasma. HMGB1 probably has the most momentum presently for use as a biomarker in the clinical care of lupus patients—especially in the context of nephritis—but confirmation in additional patient populations is needed.

There are still several gaps in our understanding of how NETs may potentially trigger autoimmunity. First, there still does not seem to be an answer to the question of whether all NETs are created equal. Replicating the proteomics data for PMA-induced NETs (Urban et al., 2009) in other systems, such as NETs spontaneously released by lupus neutrophils (Villanueva et al., 2011), or NETs triggered by MSU crystals, seems desirable and would surely generate new hypotheses regarding the potential for organ damage, and the interplay between innate and adaptive immunity.

Along these same lines, there is also still much work to be done to understand potential triggers of sterile NETosis in the rheumatologic diseases. In SVV, anti-PR3 and anti-MPO autoantibodies have been suggested as possible triggers (Kessenbrock et al., 2009), while type I interferons as well as anti-LL37, anti-RNP, and anti-double-stranded DNA autoantibodies may play a role in SLE (Garcia-Romo et al., 2011; Lande et al., 2011; Villanueva et al., 2011). These concepts await confirmation by other investigators and in animal models.

Next, there is still no validated biomarker for enhanced NETosis *in vivo*. Quantifying a circulating protein may be inferior to the strategies described for MPO (Kessenbrock et al., 2009; Caudrillier et al., 2012) and LL37/cathelicidin (Lande et al., 2011) that identify and quantify that protein in complex with DNA.

Finally, the clinical studies described above typically rely on commercial assays for the detection of autoantibodies. As will be discussed in more detail below, NETs are an attractive milieu for post-translational modifications (Liu et al., 2012), and it may take a more refined look at autoantibodies (and their specificities) to prove relevant clinical correlations, should they exist.

## Modified NET proteins as autoantigens

### Posttranslational modifications (PTMs)

PTMs are chemical alterations of a protein by the addition of biochemical functional groups (such as acetate, methyl, phosphate, lipids, and carbohydrate moieties; see Table 2), that change the chemical nature of an aminoacid (e.g., arginine > citruline) or by altering the secondary structure of the polypeptide (e.g., di-sulfide bonds). Such modifications orchestrate a variety of specific functions such as unraveling of chromatin, signaling, cell–cell recognition/communication, and enzyme activation/inactivation. Therefore, it is important to examine whether proteins externalized on the NETs undergo specific PTMs, and whether exposition of modified proteins can circumvent tolerance and promote the development of autoimmune syndromes in predisposed individuals. In this section, we will review the PTMs already reported in NET proteins.

**Table 2:** **Posttranslational modifications**.

**Modification**	**Residues modified**	**Function/notes**
Acetylation	Lys	Protein stability, DNA regulation
Deimination	Arg	Transcription
Disulfide bond formation	Cys	Protein stability, inter- intra- molecular crosslink
Glycosilation (N-, O-linked)	Asn (N-linked)	Cell–cell recognition, signaling
	Ser (O-linked)	
Methylation	Lys, Arg	Gene regulation
Nitration	Tyr	Oxidative damage during inflammation
Phosphorylation	Tyr, Ser, Thr	Activation/inactivation, signaling
Ubiquitination	Lys	Signaling, degradation

The scaffold and most abundant proteins in the NETs are histones. They comprise about 70% of the proteins associated to chromatin fibers released during NETosis to the extracellular space (Urban et al., 2009). Nucleosome is the fundamental unit of the chromatin and it is composed of two copies of each of the core histones (H2A, H2B, H3, and H4) (Luger et al., 1997). The unstructured N-terminal tail of this proteins undergoes a series of modifications, important for their role during transcription, condensation, and decondensation of the DNA. Although detection of PTMs can be a challenge, today's armamentarium includes mass spectrometry with or without proteomic analysis, and immunoblot against most common modifications (e.g., methylation, acetylation, and ubiquitination). Liu and collegues reported a series of PTMs in NETs' histones isolated from H_2_O_2_-stimulated human neutrophils and from two neutrophil-like cell lines stimulated with H_2_O_2_, TNF, LPS, Ionomycin, or PMA (Liu et al., 2012) (Table 3). Methylation of histone H4K20 (mono-, di-, and tri-methyl), acetylation of histone H4K5 and H4K16 and citrullination of histone H3 and H4 increased upon stimulation with H_2_O_2_, when compared with unstimulated conditions. The same study reported that SLE sera reacted preferentially to unmodified histone H2B and acetylated H2BK12 and K20 peptides, although a subset reacted to citrullinated histone H3 (Liu et al., 2012). In addition, autoantibodies against acetylated histone H2B tail, histone H4, histone H3K27Me3, citrullinated H3 and H4 and ubiquitination of H2A have been reported in SLE patients (Suzuki et al., 1994; Dieker et al., 2007; Van Bavel et al., 2009, 2011; Liu et al., 2012). Histone epitopes are proposed as clinically important autoantigens in SLE, RA and other autoimmune diseases (Monestier et al., 2000; Robinson et al., 2002; Van Bavel et al., 2011). Indeed, Liu and colleagues found that many of the relevant SLE autoantigens were contained in NETs (Liu et al., 2012). As mentioned above, autoantibodies generated in Felty's syndrome bind preferentially to deiminated histones, in particular to histone H3 (Dwivedi et al., 2012) and sera from these patients binds to LPS-generated NETs (Dwivedi et al., 2012). These observations further support that NETs can be a source of modified autoantigens associated with autoimmunity. Future research directions will need to focus on whether “sterile” stimuli specific for certain autoimmune diseases can induce specific PTMs in proteins externalized in the NETs, and whether these specific modifications can preferentially trigger certain chronic inflammatory processes. PTMs of various cellular proteins may trigger formation of neoantigens with the capacity to induce adaptive immune responses (Rosen and Casciola-Rosen, 1999). Despite that purified NETs failed to exacerbate autoimmune phenotypes in certain strains of mice (Liu et al., 2012), it is possible that priming factors or second signals are needed to break tolerance in the presence of aberrant NETosis. These factors may vary from disease to disease and could include, in the case of SLE, type I IFNs (Baechler et al., 2003; Bennett et al., 2003; Banchereau and Pascual, 2006), other cytokines or specific environmental insults. Indeed, a high “interferon signature” in SLE is associated with high titers of autoantibodies against histones and other nuclear proteins that may be externalized during NETosis (Baechler et al., 2003; Bennett et al., 2003). It has also been reported that type I IFNs can potentiate production of NETs (Martinelli et al., 2004).

**Table 3:** **Posttranslational modifications in NETs**.

**Source of NETs**	**Stimulation**	**Enriched PTMs found in NETs**			**Affected PTMs in NETs**		
		**Acetylation**	**Citrullination**	**Methylation**	**Acetylation**	**Citrullination**	**Methylation**
Human peripheral	Hydrogen peroxide	H4K5Ac	H3Cit(2,18,17)	H4K20Me1/2/3	H3K9Ac		
neutrophils		H4K16Ac	H4Cit3				
ATRA/GM-CSF	Hydrogen peroxide		H3Cit(2,8,17)	H3K9Me2	H2BK12Ac		H3K36Me2
differentiated murine	Ionomycin		H3Cit26	H3K27Me1/2/3	H3K9Ac		H4K20Me2
EPRO cells	LPS		H4Cit3	H4K20Me1/2/3	H3K27Ac		
	PMA				H4K16Ac		
	TNF				H3R17Me2(a)		
					H4R3Me2(s)		
ATRA differentiated	Hydrogen peroxide			H3K27Me1/2/3	H2BK12Ac	H3Cit(2,8,17)	H3K36Me2
human leukemia	LPS				H3K9Ac	H3Cit26	H4K20Me2
HL-60 cells	TNF				H3K27Ac		H3R2Me2(a)
							H3R17Me2(a)
							H4R3Me2(s)

Abbreviations *K* *lysine* *R* *arginine* *Me* *methyl* *Ac* *acetyl* *Cit* *citrulline* *(a)* *asymmetric* *(s)* *symmetric* *TNF* *tumor necrosis factor* *EPRO* *early promyelocyticcell line* *ATRA* *all-trans retinoic acid* *PMA* *phorbol 12-myristate 13-acetate* *LPS* *lipopolysaccharide*.

While most of the PTMs are often associated with reversible events involved in signal transduction, deimination (arginine to citrulline conversion)—catalyzed by a family of enzymes named peptidylarginine deiminases (PADs)—is not reversible. The presence of this atypical aminoacid (citrulline), not encoded by the genome, plays an important role during NET formation since PAD4-deficient mice suffer of impairment in NETs formation (Li et al., 2010). Studies have suggested that changes in the polarity of the aminoacid (positive to neutral) can play putative roles in the generation of autoimmune responses (Vossenaar et al., 2003; Neeli et al., 2008). High levels of PADs have been described in the central nervous system of multiple sclerosis (MS) patients and animal models of inflammatory demyelinating diseases (Mastronardi et al., 2006). Strong evidence supports a pathogenic role for citrullinated autoantigens and the immunological response to them, in RA (Schellekens et al., 2000; Suzuki et al., 2003; Lundberg et al., 2005; Foulquier et al., 2007; Duskin and Eisenberg, 2010). As histone citrullination appears to be an important phenomenon in NET formation, it remains to be established if and how this modification may promote loss of tolerance or the development of deleterious immune responses (Hirsch, 1958; Neeli et al., 2008; Li et al., 2010). Further, citrullination of other peptides that have been described present in the NETs may alter their functionality. This is the case of LL37, present in the NETs and recently found to be substrate of citrullination *in vitro* by both PAD2 and PAD4 (Lande et al., 2007, 2011; Kilsgard et al., 2012). Indeed, LL37 can be citrullinated in 3 or 5 sites and the degree of modification dictates the activity and stability of the peptide (Kilsgard et al., 2012). Indeed, LL37 (5Cit) is more chemotactic to PBMCs and more pro-inflammatory compared to LL37 (3Cit) or unmodified LL37. Thus, immunoregulation can be induced by specific PTMs that occur during NETosis. Considering that citrullination by PAD4 is essential for the generation of NETs, allowing chromatin to decondense and be released during NETosis (Wang et al., 2009; Li et al., 2010), it will be important to determine whether other proteins are citrullinated or otherwise modified in the NETs, besides histones, and in the role of these additional modifications in the regulation of inflammation and adaptive immune responses.

### Autoantigens generated by proteolytic cleavage

Another process to be considered in the generation of neoantigens is proteolytic cleavage, the process of breaking the peptide bond between two residues in a protein. Enzymes such as peptidases and proteases carry out this process and generate fragments involved in cell signaling or activation of a zymogen, the inactive form of an enzyme. Proteins can be cleaved as a result of intracellular processing. As mentioned above, NE and MPO are important during NET formation (Papayannopoulos et al., 2010). Indeed, NE can translocate into the nucleus and partially and specifically degrade histones to promote nuclear decondensation (Papayannopoulos et al., 2010). Modified or unmodified fragments of histones generated by partial cleavage could potentially be recognized as neoantigens by B- and T-cells, thereby generating autoantibodies against them. Indeed, SLE autoantibodies can recognize peptides of five aminoacids (Pro-Glu-Pro-Ala-Lys) or more and other peptides containing modifications such as methylation or acetylation, in the case of antibodies against histone H2B, using an elegant silico-based peptide array that contain every possible overlapping peptide sequence in a linear fashion against H2B (Price et al., 2012). Although, the work focused mainly on histone H2B, it shows an innovative and powerful tool to define minimum epitopes for recognition by the adaptive immune system. It remains to be fully characterized and tested whether histone fragments generated by NE during NETosis can serve as autoantigens or resemble epitopes that can be recognized by the immune system.

Some important questions remain to be answered. First, if NETs are a main source of autoantigens, how can we account for the variability in autoantibody responses among the various autoimmune diseases and among individuals with the same autoimmune condition? We may consider that not all proteins in the NETs are equally exposed. Some epitopes can be uncovered by the help of chemical agents or other molecules. This is the case of the study mentioned above regarding PTU-induced vasculitis and the development of insoluble NETs by this drug (Nakazawa et al., 2012). In this case, the high similarity of PTU to a nitrogenous base and the presence of thione group (≥S) in its structure may contribute to the high affinity to create inter and intra-molecular bonds with other sulfhydryl groups (-SH) in the NETs. Those reactions can be catalyzed by free radicals and oxidative species during NETosis, creating a compact conformation of the NETs that is insoluble. Indeed, conformational changes in the structure of the NETs may expose epitopes, such as MPO, that were not exposed in NETs in the absence of drug exposure, thereby triggering the generation of autoantibodies. Whether induction of aberrant NET structure may be one of the key mechanisms implicated in drug-induced lupus remains to be established. It will also be important to examine whether NETs triggered by specific conditions present in certain autoimmune diseases (ANCAs, anti-RNP antibodies, IFNs, etc.) induce different rearrangement of the chromatin and/or other modifications that promote specific protein content of the NETs and/or changes in their structure.

Taken together, exposure of altered proteins on the NETs, either by PTMs, proteolytic cleavage or specific environmental stimuli (e.g., drugs) in the context of an underlying pro-inflammatory milieu could promote deleterious consequences for the host. In addition, patients with deficiency in the clearance of NETs, such as that described in patients with SLE (Hakkim et al., 2010), may confer enhanced, persistent exposition of NETs and associated proteins that may promote generation and perpetuation of autoimmune responses. In this scenario, autoantibodies against specifically modified antigens could serve as prospective biomarkers for autoimmune diseases beyond RA.

## Conclusions

NETs may represent an important source of neoantigens, where PTMs and proteolytic cleavage of proteins externalized in the NETs could promote the generation of autoantibodies in predisposed individuals. Indeed, the generation of autoantibodies to modified autoantigens has been described, suggesting a link between PTMs and autoimmunity. While NETs are unlikely to be the only source of autoantigens in SLE and other autoimmune/inflammatory diseases, the combination of PTMs derived from NETs and inflammatory molecules that may act as priming factors, represent an attractive milieu for the loss of tolerance and/or the activation of deleterious innate and adaptative immune responses.

### Conflict of interest statement

The authors declare that the research was conducted in the absence of any commercial or financial relationships that could be construed as a potential conflict of interest.
